# HipSim — hip fracture surgery simulation utilizing the Learning Curve–Cumulative Summation test (LC-CUSUM)

**DOI:** 10.1080/17453674.2020.1777511

**Published:** 2020-06-15

**Authors:** Jan Duedal Rölfing, Rune Dall Jensen, Charlotte Paltved

**Affiliations:** a Corporate HR, MidtSim, Central Denmark Region; b Department of Clinical Medicine, Aarhus University; c Department of Orthopaedics, Aarhus University Hospital, Denmark

## Abstract

Background and purpose — Virtual reality simulation of hip fracture surgery is available for orthopedic residents nationwide in Denmark. Summative assessment of learning applying the learning curve cumulative summation test (LC-CUSUM) has not been utilized in orthopedic simulation training. The strength of the LC-CUSUM is that it assumes incompetency and signals competency based on solid statistics. We investigated the LC-CUSUM characteristics of novices stepwise mastering the simulated dynamic hip screw (DHS) procedure.

Material and methods — 32 1st-year orthopedic residents participated in HipSim and its 3 subsequent LC-CUSUM evaluations: placing a Kirschner wire, placing a Kirschner wire in different patients, and performing the entire DHS procedure in different patients. The career status of the participants, i.e., still working in orthopedics or in another specialty was recorded ≥ 2 years after participation and associated with the simulation performance (passed/failed).

Results — 13/14 participants passing HipSim according to LC-CUSUM were still working in orthopedics, while 9/18 participants failing HipSim had quit orthopedics at ≥ 2 years follow-up. The simulator-generated feedback did not statistically significantly differ between the groups.

Interpretation — LC-CUSUM and its summative pass/fail assessment of each simulation was feasible in this formative simulation program. Clinical educators can be reassured that participants passing HipSim are likely to continue to 2nd–5th year of residency, while failing HipSim should raise concerns and trigger career counselling and clinical supervision. The motivational aspect of LC-CUSUM pass/fail assessment when designing formative simulation training warrants further research.

The high mortality rate after hip fractures is caused by a multitude of factors including comorbidities, the effects of immobilization due to the fracture, and suboptimal surgical treatment and rehabilitation (Brauer et al. 2009, Röck et al. 2019). Ideally, a hip fracture operation will allow the patient to immediately fully weight-bear. Intraoperative adverse events and insufficient stabilization of the fracture significantly prolong recovery and worsen the prognosis. Thus, high-quality surgical training is often emphasized as key.

Virtual reality (VR) simulation training has been introduced to address the issue of training, and previous studies have demonstrated that VR training can ensure basic proficiency (Pedersen et al. 2014, Gustafsson et al. 2019). Consequently, VR training in hip fracture surgery is available to 1st-year orthopedic residents in Denmark at an early stage of their orthopedic career and before applying for and enrolling in the 2nd–5th years of orthopedic residency.

Since the Learning Curve–Cumulative Summation test (LC-CUSUM) was first described by David Biau et al. (2008), it has gained popularity in monitoring skill acquisition and quality control of surgical performance when learning new procedures. In orthopedics, LC-CUSUM has been applied to signal competency of the surgeon in a multitude of procedures, e.g., spinal laminectomy, and total hip and knee replacement surgery (Lee et al. 2014, Zhang et al. 2014, Park et al. 2019). We present the 1st study applying LC-CUSUM in orthopedic simulation training. The major strength of the LC-CUSUM is that it assumes incompetency and can signal competency based on solid statistical methods. Consequently, LC-CUSUM could prove beneficial to determine thresholds in simulation-based training, e.g., if the null hypothesis of an inadequate performance is rejected, the learner can be allowed to continue the learning process in the clinical setting (summative assessment of learning). If this holds true, simulation-based LC-CUSUM training programs can facilitate the education of young surgeons, who can safely learn to master the initial steps of the learning curve without imposing risk on patients.

We describe the LC-CUSUM characteristics of DHS simulation training (HipSim) of 1st-year orthopedic residents and associate the results with the participants’ career status after ≥ 2 years follow-up.

## Methods

### Simulator

**Figure 1.**
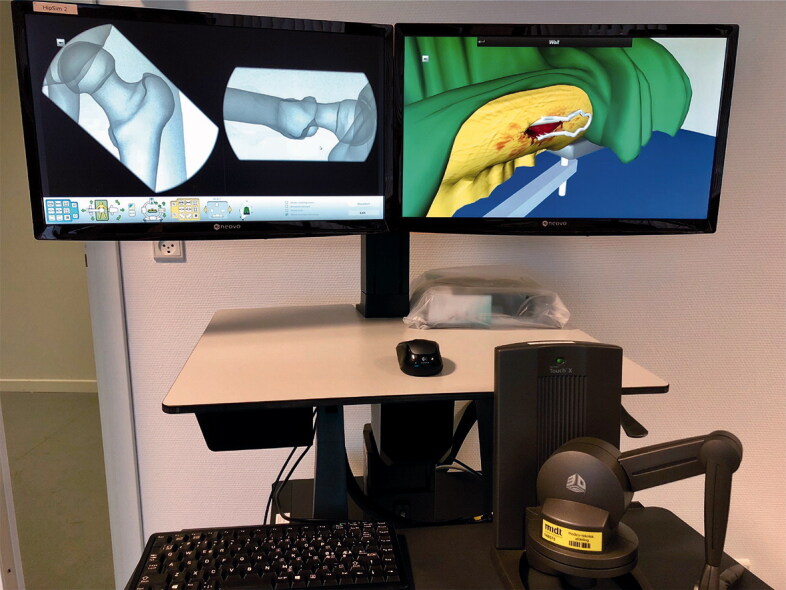
The haptic-feedback virtual reality simulator (TraumaVision, Swemac Simulation AB, Sweden).

The construct validity of the virtual reality (VR) simulator (TraumaVision, Swemac Simulation AB, Sweden) has been established, i.e., the simulator performance correlates with surgical experience (Pedersen et al. 2014, Akhtar et al. 2015, Gustafsson et al. 2019). Notably, the simulator can discriminate between novices (< 10 DHS), intermediates, and experts (Akhtar et al. 2015). The simulator provides the opportunity to train the basic skills required for the clinical DHS procedure, e.g., hand–eye coordination and the ability to work in 3 dimensions based on 2-dimensional visual information and haptic feedback (Figure 1).

### Participants

The DHS simulation training was available for all 1st-year orthopedic residents who had performed fewer than 10 hip fracture-related surgeries under supervision (cannulated screws, DHS, intramedullary nails, hemi-/total hip arthroplasties) from Central Denmark Region and Northern Denmark Region from November 2016 until January 2018. All departments have written agreements to send all their residents in fulfilment of the < 10 procedure criterion, thus all residents are expected to participate in HipSim training, which takes place during working-hours. Consequently, selection bias is unlikely.

Retrospectively, we divided the participants into 2 cohorts depending on whether or not they were pursuing a career in orthopedics in January 2020 (e.g. enrolment in 2nd–5th year of orthopedic residency or orthopedic PhD program).

### Simulation program and LC-CUSUM design

The simulation was designed as a time-dispersed mastery-learning program with 2–3 training days of a maximum 4 hours of training per day.

The DHS simulation program introduced participants stepwise to the surgical procedure. At competency level 0 (CL0), participants had to become acquainted with the simulator and master the key step of placing a Kirschner wire (K-wire) in the fractured left hip of the same patient repetitively. Subsequently, the compexity was increased at CL1 and CL2. Clinical variability was introduced at CL1 with up to 24 different scenarios (different patients and fracture patterns), and the entire DHS procedure was introduced at CL2. Here, the participants also had to choose the optimal DHS angle for the given patient, insert the K-wire, ream and insert the sliding screw, and fixate the plate to the femoral shaft with a single bicortical screw.

Advancement to the next CL was granted when LC-CUSUM signaled competency.

### Pass/fail criteria

In order to apply the LC-CUSUM to any procedure, the procedure has to be classified as “passed” or “failed”. Hence, passing/failing criteria had to be established: a tip apex distance (TAD) of more than 20 mm is a scientifically validated predictor of failure of internal fixation (Baumgaertner et al. 1995, De Bruijn et al. 2012). Other failing criteria were, for instance, more than 3 attempts to place the K-wire appropriately or the breach of cortical bone with either the K-wire or the reamer.

Besides the immediate simulator-generated feedback of passing/failing a procedure, secondary feedback data included the passing criteria: TAD ≤ 20 mm, center–center or center–inferior placement, no violation of cortical bone, and no more than 3 attempts to place the K-wire. For a complete list of passing/failing criteria for CL0 (= CL1), and CL2 respectively, please see Figure 2, Supplementary data. Furthermore, information regarding the total time, fluoroscopy time, and number of radiographs were provided, but did not influence whether the procedure was passed or failed.

### Learning Curve–Cumulative Summation test (LC-CUSUM)

The statistical design of the LC-CUSUM test was based on personal correspondence with D. Biau. The adequate performance level was set at 10% failure and the equivalence zone (delta) at 5%, i.e., the acceptable deviation from adequate performance. An in-control limit, h = 0.74, was chosen to give a probability of declaring competency if the participant’s true performance is adequate (true positive, akin to power) of 91% and a probability of declaring competency if the participant’s false performance is inadequate (false positive, akin to type I error) of 12% over 50 procedures. The score gain for a successful procedure was +0.057 and the loss for a failure was –0.405.

In order to make LC-CUSUM easier to comprehend it was slightly modified by setting the in-control limit (h) to 13, the score gain for a successful procedure to +1 and the loss for a failure to –7. Consequently, 13 successful procedures in a row will reject the null hypothesis of inadequate performance at the given CL 0, 1, or 2, respectively. Thus, a minimum number of 39 procedures was required to complete all 3 CLs, while the maximum number of procedures was capped at 50 procedures for each level.

The LC-CUSUM design dictates that a single failure during the learning process causes the subtraction of 7 points on the LC-CUSUM score. This may seem rather punitive, as a single failure thus requires up to 7 successful procedures afterwards in order to regain the same LC-CUSUM score as before the failure. However, it is statistically required to be able to ensure the high standard of adequate performance among successful participants. Furthermore, it should be highlighted that the responsiveness of the LC-CUSUM to learning is preserved, because early failures during learning are not punished as hard as failures at later stages during the learning process, e.g., the LC-CUSUM score cannot be negative and is bound by 0–13 for CL0, 13–26 for CL1, and 26–39 for CL2 (Biau et al. 2008).

### Statistics

Data are presented as mean (95% confidence interval, CI) or median (min–max). Both a chi-square test and Fisher’s exact test were used to analyze the 2 x 2 contingency table (passed/failed x orthopedics/other). 1-way ANOVA with uncorrected Fisher’s least significant difference test for multiple group comparison was applied to test for statistically significant different means between the three groups (passed orthopedics, failed orthopedics, and failed other specialty) according to Table. A p-value ≤ 0.05 was considered statistically significant; however, predominantly CI is given instead of p-values according to Acta Orthopaedica author guidelines.

### Ethics, funding, and potential conflicts of interest

Ethical approval (no. 251/2016) was granted by the Ethical Committee, Central Denmark Region on November 23, 2016. Funding was granted by the Central Denmark Region. No external funding was obtained. The current HipSim version of TraumaVision, Swemac Simulation AB, Sweden was programmed by engineers from Swemac Simulation AB according to suggestions by JDR. No direct or indirect financial contributions were received; thus, all authors declare no conflicts of interest.

## Results

32 1st-year orthopedic residents fulfilled the inclusion criteria (< 10 hip fracture surgeries before HipSim participation and subsequently at least 2 years follow-up). 22 participants were enrolled in an orthopedic residency or an orthopedic PhD program at latest follow-up. 10 participants changed to another medical specialty and were no longer working within orthopedics at latest follow-up.

There was no statistically significant difference in the male/female ratio, age, and hand dominance between 1st-year orthopedic residents who continued or quit a career in orthopedics. Furthermore, there were no statistically significant differences in these characteristics between participants who passed or failed HipSim according to LC-CUSUM.

**Figure 3.**
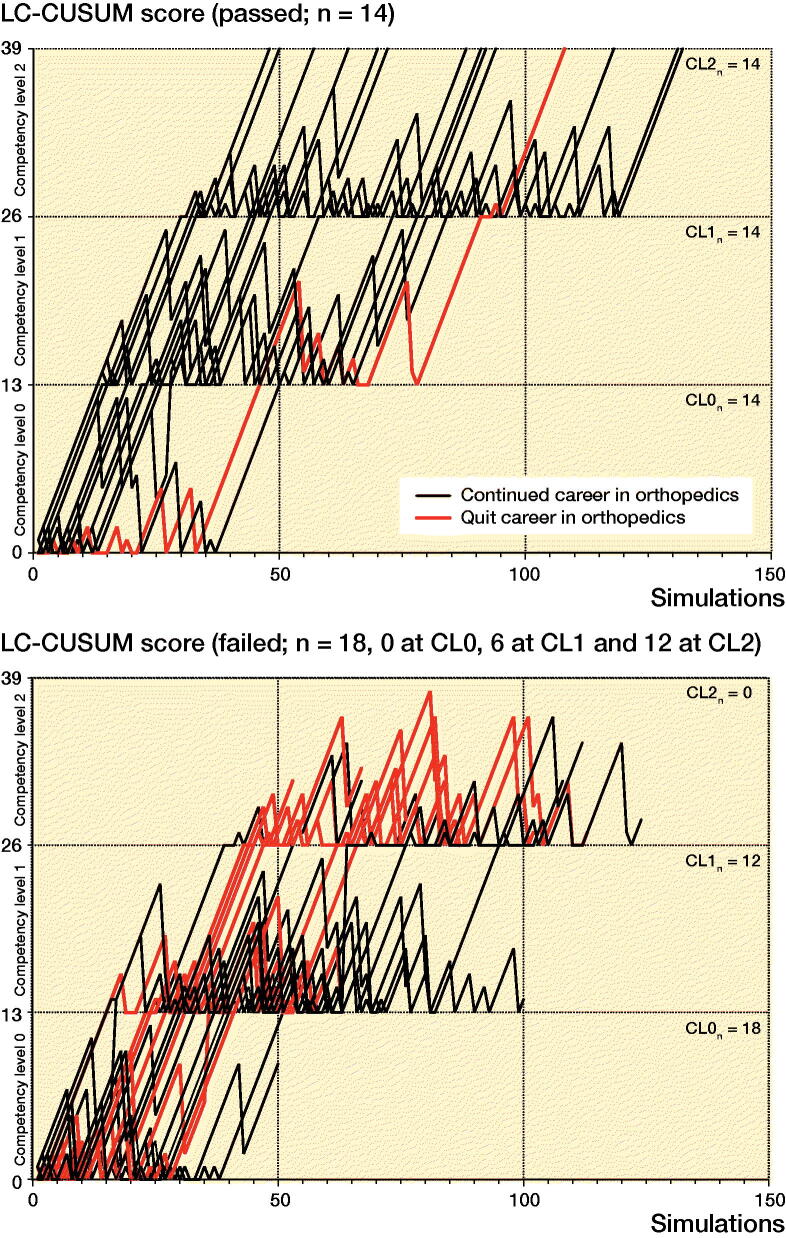
Learning Curve–Cumulative Sum (LC-CUSUM) chart illustrating passing (+1 LC-CUSUM score) and failing (–7 LC-CUSUM score) of each simulation of the 14 participants passing competency levels 1–3 (CL; upper panel) and 18 participants failing at one of the competency levels. 13/14 passed and 9/18 failed participants still pursued a career in orthopedics at ≥ 2 years’ follow-up.

14/32 participants reached an LC-CUSUM score of 39 and thereby passed the simulation program according to the LC-CUSUM criteria (max. 50 simulations at each of the 3 CLs to obtain an LC-CUSUM score of 13 and advance to the next CL; Figure 3).

13/14 of the participants who passed the simulation program are currently enrolled in an orthopedic residency program, while only 9/18 of the participants who failed the LC-CUSUM test still pursued a career in orthopedic surgery at 2 years follow-up (p = 0.02). Hence, the positive predictive value of passing HipSim and continue to the 2nd–5th-year orthopedic residency was 93% (CI 69–100) and the negative predictive value was 50% (CI 29–71). Accordingly, the sensitivity was 59% (CI 39–77) and the specificity was 90% (CI 60–99).

For details regarding the number of simulations, simulation time, use of fluoroscopy and radiographs, and tip apex distance, see Table. None of the differences in these outcome measures reached statistical significance. However, a higher number of simulations (mean difference 32 [CI -3–67]) was noted among failed participants still working in orthopedics at follow-up vs. failed participants who had quit orthopedics at 2 years follow-up.

## Discussion

Overall less than half of all participants (14/32) passed HipSim according to the LC-CUSUM criteria. The positive predictive value of pursuing a career in orthopedics was 93%. However, the majority of participants failing HipSim, but still pursuing a career in orthopedics more than 2 years after the simulation program, were persistent and continued simulation training until they reached the predefined passing LC-CUSUM score of 39, but with more than the maximum allowed simulations, while 9/10 of participants working in other medical specialties 2 years after training failed according to LC-CUSUM.

In contrast to previous studies with the simulator, this study is the first to include clinical variability in the simulation program. Previous studies have considered the validity of the simulator and explored learning curves of repetitively operating on the same side of a single patient (Pedersen et al. 2014, Akhtar et al. 2015, Sugand et al. 2015, Gustafsson et al. 2019). We strongly believe that the introduction of clinical complexity is essential to exploit the full learning potential of the VR simulation program.

### Implications for orthopedic simulation training of the summative LC-CUSUM (pass/fail) assessment

One of the participants failed the LC-CUSUM (within the maximal number of 50 procedures per competency level), but was allowed to continue simulation training and performed no less than 239 procedures before reaching the LC-CUSUM score of 3 × 13 = 39 (Figure 4, see Supplementary data). The learning curve of this participant underlines the aim of HipSim to educate and ensure acquisition of basic technical skills rather than providing a summative (pass/fail) assessment of the participant. This participant enrolled in an orthopedic residency program and continuously receives excellent ratings of his/her clinical performance including osteosynthesis of hip fractures. Thus, HipSim is a formative simulation-based training program, but utilizing the strength of LC-CUSUM with a summative evaluation of each simulation. Consequently, the results of HipSim should not and cannot be used as a summative assessment of the participant’s capabilities to become a competent surgeon (Aucar et al. 2005, Strandbygaard et al. 2017).

9/18 of the participants failing HipSim at an early stage of their 1st year in orthopedics did not continue their career in orthopedics and did not enroll in 2nd–5th year residency or an orthopedic PhD program. These results may prove useful for orthopedic educators in the future. Passing HipSim may be a confounder for the determination of the participant to become an orthopedic surgeon. Hence, passing the LC-CUSUM simulation program may reassure clinical supervisors of the potential of their residents, whereas failing the simulation program may draw the educators’ attention to career counselling in order to help their first-year residents in their decision-making on whether to pursue a career in orthopedics or not. This hypothesis is supported by the observation that some of the junior doctors currently employed in other specialties did not return for the second training day compared with the more determined participants, for instance the orthopedic resident who participated on 3 days and simulated 239 procedures compared with a mean of 79 (CI 61–89) simulations for participants failing HipSim and quitting orthopedics. Furthermore, we speculate as to whether participants with the career plan to work as general practitioners or within emergency medicine may have applied for the 1st-year residency in orthopedics with the purpose of acquiring an insight and clinical evaluation skills without an earnest interest in the surgical procedures or surgical simulation. However, these hypotheses require further scientific investigations.

### Perspectives regarding unsolved issues in medical education

The main purpose of hip fracture simulation programs is to advance novices to become pre-trained novices with an accelerated learning curve when starting to learn to operate in the clinical setting. To our knowledge no transfer studies have investigated this issue regarding hip fracture surgery. However, a randomized controlled trial investigated the effect of VR training of total hip arthroplasty and reports the transferability of the acquired technical skill to a cadaver lab (Hooper et al. 2019). Furthermore, the ability of the participants to use their acquired competencies in different, related hip fracture procedures such as cannulated screws and intramedullary nailing should be tested. Notably, the transferability of technical skill from one surgical procedure to another has been a matter of debate (Van Sickle et al. 2006, Akhtar et al. 2016).

From a medical education point of view, future studies are needed to investigate whether a summative LC-CUSUM test promotes or hinders formative acquisition of technical competencies. The latter might be the case, because the LC-CUSUM test punishes failed simulations harshly, causing frustration (Rölfing et al. 2019). Thus, it is relevant to investigate if LC-CUSUM should be included or if other more motivating learning strategies should be applied in simulation-based training programs.

### Need for continued clinical supervision and monitoring

In the present study, none of the participants had flawless performance, i.e., reaching an LC-CUSUM score of 39 without any mistakes. Furthermore, the vast majority of participants made mistakes not only at early stages of the learning curve, but also at later stages. This highlights the strength of the LC-CUSUM as it visualizes these late failed procedures (Figure 1). In the clinical setting, orthopedic residents in Scandinavia are often permitted to operate under no or limited supervision after 3–5 successful supervised procedures only. In our study, even participants with 12 consecutive, successful procedures made critical mistakes and failed the next procedure, which underlines the need for continued clinical supervision. Furthermore, the number of simulations and total simulation time was comparable between groups. Thus, competency cannot be declared based on the number of procedures or the time in training (Gustafsson et al. 2019).

This important take-home message and the ability of the LC-CUSUM to monitor successful procedures before signaling competency with predefined statistical certainty was also appreciated in clinical studies regarding spinal laminectomy and acetabular cup placement in total hip arthroplasty (Lee et al. 2014, Park et al. 2019).

Finally, we agree with the notion that simulation training can help to acquire the skills needed to perform the surgical procedure, such as hand–eye coordination, and the ability to work in 3 dimensions based on 2-dimensional visual information and haptic feedback (Akhtar et al. 2016, Marcheix et al. 2017). VR simulation is currently neither suited to teaching the participants the entire procedure including reposition of the fracture and soft-tissue handling nor does it encompass the overwhelming complexity of performing the procedure in the operating theatre. However, the major benefit of HipSim resides in learning the skills needed to perform the DHS surgical procedure, including the sequence of surgical steps. Thus, HipSim-certified pretrained novices are prepared to continue the learning process under close supervision in the operating theatre. The orthopedic community is becoming increasingly aware and appreciates this beneficial aspect of simulation training (Morgan et al. 2017, Kalun et al. 2018, Atesok et al. 2019, Gustafsson et al. 2019).

In conclusion, LC-CUSUM and its summative pass/fail assessment of each simulation was feasible in this formative simulation program. Clinical educators can be reassured that participants passing HipSim are likely to continue to the 2nd–5th years of residency, while failing HipSim should raise concerns and career counselling and close clinical supervision seem to be appropriate measures. The motivational aspect of LC-CUSUM pass/fail assessment when designing formative simulation training warrants further research.

**Table:** 

	Passed (n = 14)		Failed (n = 18)	
	Career in	Career in	Career in	Career in
	orthopedics	other specialty	orthopedics	other specialty
	n = 13	n = 1	n = 9	n = 9
Training sessions (days) ^a^	2 (1–3)	1	2 (1–3)	2 (1–3)
Total simulation time (min.)	196 (152–241)	157	186 (119–252)	170 (123–217)
Mean time of last 3 simulations (s)				
at CL0	66 (47–85)	52	71 (58–86)	77 (59–95)
at CL1	77 (60–94)	85	66 (37–95)	109 (76–142)
at CL2	206 (168–244)	230	199 (82–315)	208 (135–282)
Mean total simulations (n)	89 (73–106)	108	109 (66–150)	79 (61–96)
Simulations at CL0 (n)	23 (16–31)	46	30 (22–49)	32 (22–41)
Simulations at CL1 (n)	33 (24–41)	45	38 (25–50)	29 (22–36)
Simulations at CL2 (n)	33 (25–41)	17	19 (3–35)	21 (1–41)
Mean TAD ^b^ of last 3				
simulations at CL2 (mm)	13 (12–15)	14	13 (10–17)	15 (11–19)
Mean cortical drill outside				
femoral shaft (mm)	6 (5–7)	8	8 (5–10)	7 (5–19)
Mean fluoroscopy time of last 3 simulations (s)				
at CL0	9 (2–45)	7	5 (2–18)	10 (2–42)
at CL1	13 (5–49)	23	9 (2–27)	13 (4–50)
at CL2	31 (9–55)	46	28 (11–63)	24 (8–41)
Mean radiographs of last 3 simulations (n)				
at CL0	21 (10–37)	17	24 (4–37)	21 (17–30)
at CL1	22 (7–51)	22	15 (4–28)	32 (11–37)
at CL2	36 (15–91)	26	41 (17–74)	44 (22–55)

**^a^**median (range)

**^b^**TAD: tip–apex distance

## Supplementary Material

Supplemental Material

## Supplementary data

Figures 2 and 4 are available as supplementary data in the online version of this article, http://dx.doi.org/10.1080/17453674.2020.1777511

Simulator-generated feedback of passed and failed 1st-year orthopedic residents (n = 32) subdivided into the two cohorts: career in orthopedics/career in other specialty 2 years after HipSim training. Values are mean (95% CI) unless otherwise specified
