# Supplementary material for: HipSim — hip fracture surgery simulation utilizing the Learning Curve–Cumulative Summation test (LC-CUSUM)
Source: Acta Orthop. 2020 Jun 15;91(6):669–74. doi: 10.1080/17453674.2020.1777511 (PMC8023956; doi:10.1080/17453674.2020.1777511)
Supplement: Supplemental Material [file IORT_A_1777511_SM9494.pdf]

Supplementary data

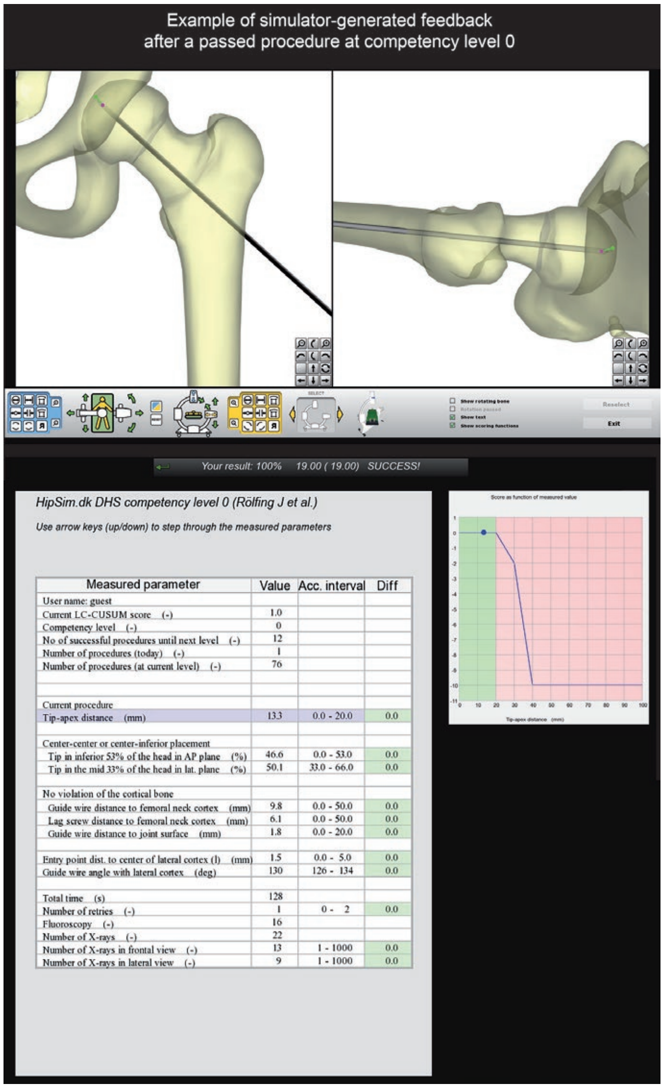

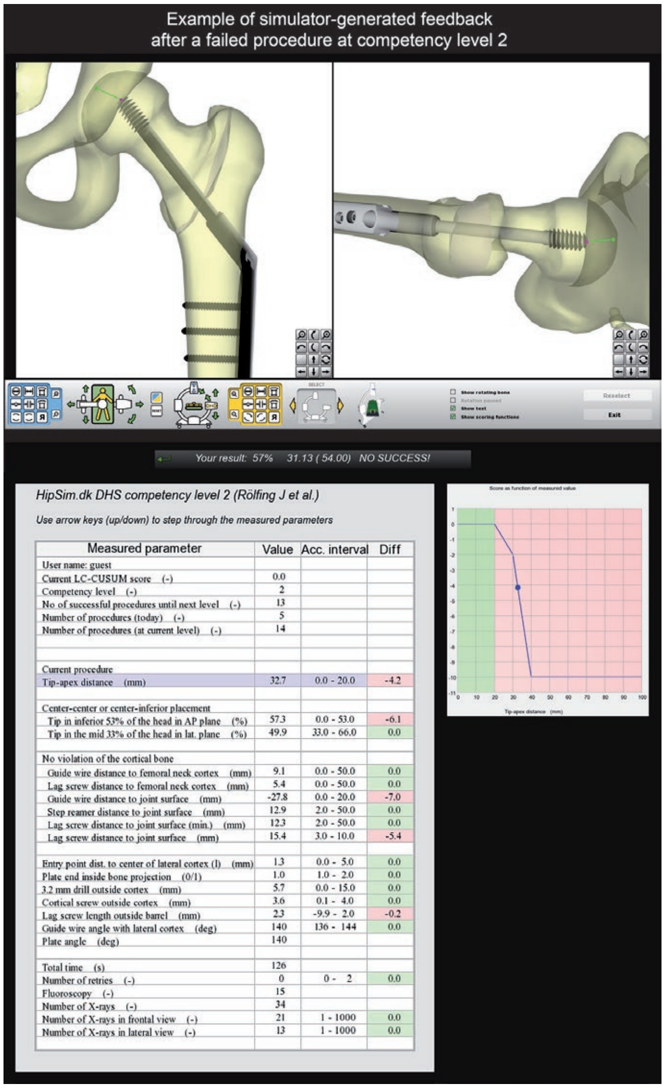

Figure 2. Passing/failing criteria of CL0 (which are equal to the passing/failing criteria of CL1) on the left, and passing/failing criteria of CL2 on the right.

 Acta Orthopaedica 2020; 91 (DOI: 10.1080/17453674.2020.1777511)

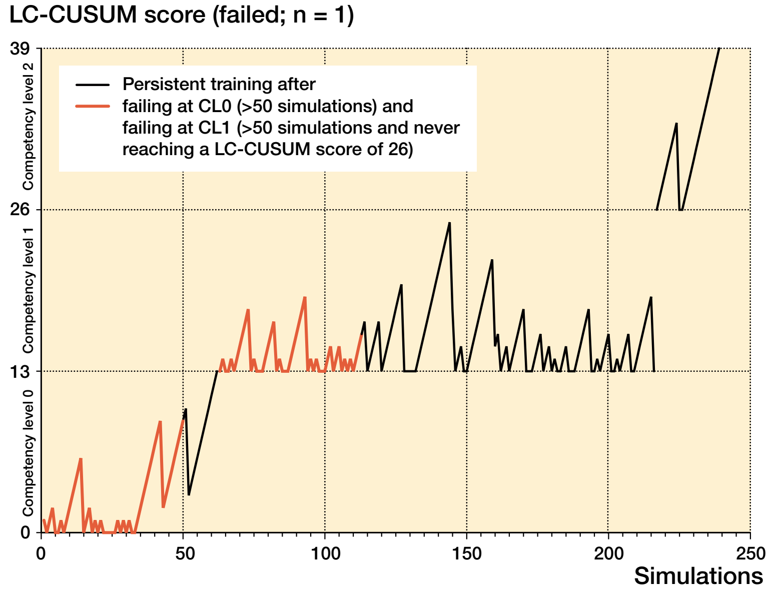

Figure 4. LC-CUSUM chart of a persistent and determined participant who failed both CL0 and CL1, but continued training performing a total of 239 simulations on 3 training days.
